# Statistical methods for predicting tuberculosis incidence based on data from Guangxi, China

**DOI:** 10.1186/s12879-020-05033-3

**Published:** 2020-04-22

**Authors:** Yanling Zheng, Liping Zhang, Lei Wang, Ramziya Rifhat

**Affiliations:** College of Medical Engineering and Technology, Xinjiang Medical University, Urumqi, 830011 People’s Republic of China

**Keywords:** Tuberculosis, Prediction, SARIMA model

## Abstract

**Background:**

Tuberculosis (TB) remains a serious public health problem with substantial financial burden in China. The incidence of TB in Guangxi province is much higher than that in the national level, however, there is no predictive study of TB in recent years in Guangxi, therefore, it is urgent to construct a model to predict the incidence of TB, which could provide help for the prevention and control of TB.

**Methods:**

Box-Jenkins model methods have been successfully applied to predict the incidence of infectious disease. In this study, based on the analysis of TB incidence in Guangxi from January 2012 to June 2019, we constructed TB prediction model by Box-Jenkins methods, and used root mean square error (RMSE), mean absolute error (MAE) and mean absolute percentage error (MAPE) to test the performance and prediction accuracy of model.

**Results:**

From January 2012 to June 2019, a total of 587,344 cases of TB were reported and 879 cases died in Guangxi. Based on TB incidence from January 2012 to December 2018, the SARIMA((2),0,(2))(0,1,0)_12_ model was established, the AIC and SC of this model were 2.87 and 2.98, the fitting accuracy indexes, such as RMSE, MAE and MAPE were 0.98, 0.77 and 5.8 respectively; the prediction accuracy indexes, such as RMSE, MAE and MAPE were 0.62, 0.45 and 3.77, respectively. Based on the SARIMA((2),0,(2))(0,1,0)_12_ model, we predicted the TB incidence in Guangxi from July 2019 to December 2020.

**Conclusions:**

This study filled the gap in the prediction of TB incidence in Guangxi in recent years. The established SARIMA((2),0,(2))(0,1,0)_12_ model has high prediction accuracy and good prediction performance. The results suggested the change trend of TB incidence predicted by SARIMA((2),0,(2))(0,1,0)_12_ model from July 2019 to December 2020 was similar to that in the previous two years, and TB incidence will experience slight decrease, the predicted results can provide scientific reference for the prevention and control of TB in Guangxi, China.

## Background

Tuberculosis (TB) is a chronic respiratory infectious disease caused by the pathogen *Mycobacterium tuberculosis*. Infected people can spread TB germs from their mouth when they cough or sneeze. After suffering from TB, if the patients are not given timely, thorough treatment, which can pose a serious threat to their health, even make them completely lost the ability to work, and the TB patients may also infect others [1]. At present, although great progress has been made around the world in the prevention and control of TB, many countries, especially in low-income and middle-income settings, are still afflicted with a chronic plague of TB with huge economies losses [2]. Moreover, TB remains one of the top 10 causes of death worldwide; it is estimated that globally there were 10.0 million new cases of TB in 2017, of which 1.3 million individuals’ deaths were directly attributable to TB, and TB has killed more people than any other infectious disease in the past few decades [2, 3]. China is one of the countries with high burden of TB, number of TB patients ranked second in the world, accounting for a quarter of the world’s patients, and about 250 thousand patients died of TB every year in China [2].

The Guangxi is a province of China, it is located in the south of China, the latitude 20°54′ ~ 26°24′ N, longitude 104°26′ ~ 112°04′ E, it covers a total area about 236,700 km^2^, with a population over 49.26 million in 2018, and is one of the Chinese provinces that is most affected by TB. From 2015 to 2017, the annual incidences (per 100,000 populations) of TB in China were 63.42, 61 and 60.53, respectively, while the annual incidences of TB in Guangxi province of China were 96.41, 86.27 and 87.86, respectively. These incidences of TB in Guangxi were much higher than that in the national level, so it is necessary to pay more attention to the prevention and control of TB in this area.

To master the regularity of infectious diseases, analyze and know the epidemic situation of infectious diseases by using the existing surveillance data, then predict the future, which can provide scientific reference for disease prevention and control. The Box-Jenkins method is a representative time series analysis and prediction method, which can take into account trend changes, periodic changes, and random disturbances in time series. It is very useful in modeling temporal dependence structure of a time series. At present, this method has been widely used in the prediction of infectious diseases, and has achieved successful prediction results, for instance, Tian C W et al. [4] forecasted monthly cases of hand-foot-mouth disease successfully in China; Wang T et al. [5] suggested that ARIMA(3,1,1)(2,1,1)_12_ model was reliable with a high validity, which could be used to predict hemorrhagic fever with renal syndrome incidence in Zibo; Myriam Gharbil et al. [6] predicted the dengue incidence in Guadeloupe based on time series analysis; López-Montenegro LE [7] predicted dengue cases in Colombia from 2018 to 2022 based on Auto-Regressive Integrated Moving Average (ARIMA) model; Zheng Y-L et al. [8] and Liao Z [9] forcasted TB incidence successfully using SARIMA model, etc. [10–17].

The incidence of TB in Guangxi is very high, but there are few related prediction studies so far. In order to do a better job of prevention and control, in the study, the prediction research was carried out. Firstly, we briefly analyzed the change trend of the TB incidence in Guangxi over the years, and then, based on the data characteristics of the TB incidence in Guangxi, China, we established the best SARIMA model for prediction. Finally, the TB incidence in the future was predicted, which can provide scientific reference for prevention and control of TB in Guangxi.

## Methods

### Data source

The data of the TB cases in Guangxi from January 2012 to June 2019 was obtained from the Guangxi center for Disease Control and Prevention, China; Population data was obtained from the official website of Guangxi Bureau of Statistics, based on the population data and the reported number of TB cases, we calculated the monthly incidence of TB (per 100,000 populations). The data used in this study is provided as Additional file 1.

### SARIMA model descriptions

The Box-Jenkins method is a famous time series prediction method proposed by Box and Jenkins in the early 1970s, it includes the ARIMA(p,d,q) model called. Autoregressive Integrated Moving Average Model, AR is auto regression, p is the number of auto regression term, MA is moving average, q is the number of moving average terms [18, 19]. If the time series contains a seasonal cycle, it is often necessary to do a seasonal difference to establish a SARIMA model, the SARIMA model with *s* observations per period, denoted by SARIMA (*p*, *d*, *q*)(*P*, *D*, *Q*)s. Generally, the standard statistical methodology to construct an SARIMA(*p*, *d*, *q*)(*P*, *D*, *Q*)s model includes four steps:

First step, data stationary test. Usually, data set needs to be divided into two subsets for model: one for training set, and the other one for testing set. The training set needs to be stationary time series. If the original training set data is not stationary, common differential or seasonal difference is required, d is the order of the ordinary difference, and D is the order of the seasonal difference. Augmented Dickey-Fuller (ADF) test can determine whether the time series was stationary, the significance level of the test is 0.05 (if the test Prob is less than 0.05, then, the data is stationary).

Second step, based on the data of stationary time series, to plot the graphs of the autocorrelation function (ACF) and partial autocorrelation function (PACF). According to the analysis of ACF and PACF, we can determine the possible values of *p*, *q*, *P* and Q, this process requires both skill and experience. Generally, more than one tentative model is chosen in this step.

Third step, to do parameter estimation and hypothesis test of all tentative SARIMA models by least square method. These model passed by the parameter test is feasible, furthermore, to do diagnostic checking of their residuals, if residuals are almost equivalent to white noises (significant level Prob> 0.05) by using the Box-Jenkins Q test, then SARIMA model has good performance. Then, to select the best SARIMA model by the Akaike information criterion (AIC) and Schwarz criterion (SC). The preferred model is the one with the lowest AIC and SC values.

Forth step, to predict the TB incidence based on the preferred SARIMA model, then, to calculate forecast accuracy indexes, such as root mean square error (RMSE), mean absolute error (MAE) and mean absolute percentage error (MAPE). Good fitting and prediction performance of SARIMA model are demonstrated with RMSE, MAE and MAPE as small as possible.

### Data processing and analysis

All analyses were performed using ArcGIS 10.4, Eviews7.2, R3.6.2 and Matlab 2012b.

## Results

**Fig. 1:**
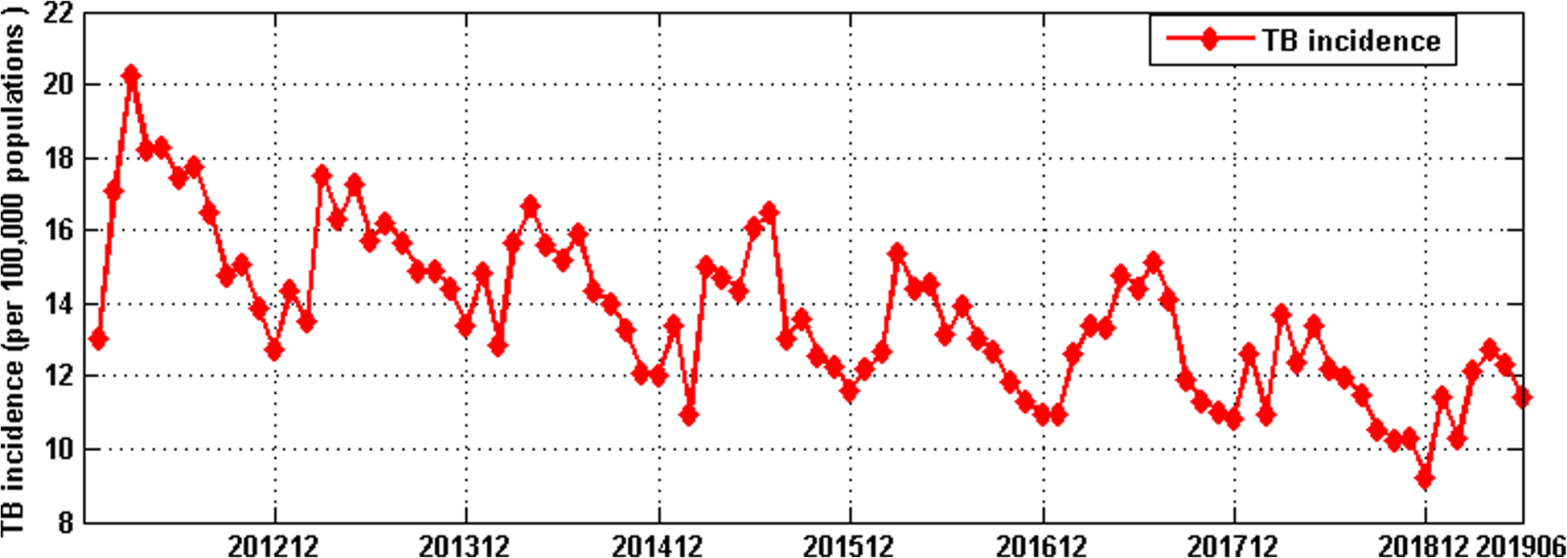
The TB incidence in Guangxi from January 2012 to June 2019

From January 2012 to June 2019, a total of 587,344 cases of TB and 879 deaths of TB were reported in Guangxi. It can be seen from Fig. 1 that the TB incidence was decreasing year by year, and there was certain seasonality. The TB incidence in the second and third quarters were higher than that in the first and fourth quarters.

**Fig. 2:**
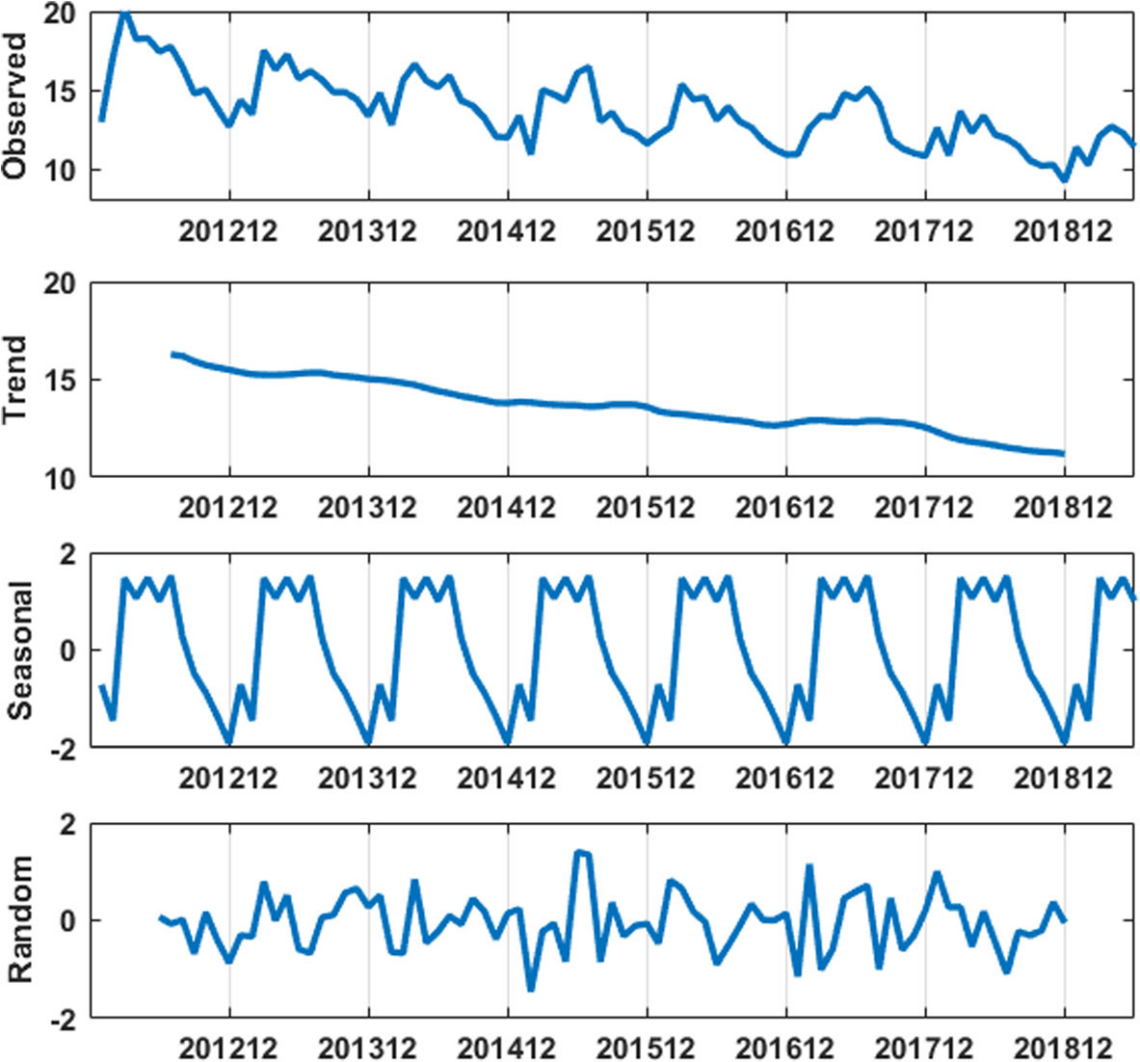
Time series decomposition of TB incidence from January 2012 to June 2019

We used R3.6.2 software to decompose TB incidence data, and found that TB incidence data have obvious seasonality, periodicity and randomness (see Fig. 2), so it is suitable to establish SARIMA model for prediction analysis.

The data from January 2012 to June 2019 was divided into two parts, the part from January 2012 to December 2018 was used to construct the SARIMA(*p,d,q*)(*P,D,Q*)_s_ model, and the other part from January 2019 to June 2019 was used to test the prediction performance of the SARIMA(*p,d,q*)(*P,D,Q*)_s_ model.

The SARIMA(*p,d,q*)(*P,D,Q*)_s_ model method requires data to be stationary, otherwise, neither of backcast or forecast of the series can be available. First, ADF was used to test the stability of original series, and the tested Prob value was 0.94 greater than 0.05, which showed that the series was not stationary. Because there was obvious seasonality in the TB incidence series in Guangxi (see Fig. 2), we did the first-order seasonal difference with period 12 on original series, and then, did ADF test of the seasonal difference data again, and the tested Prob value was less than 0.01, therefore, after the first-order seasonal difference, the data was stationary, then, d = 0, D = 1 and s = 12. The test results were shown in Table 1.

**Table 1:** The ADF tests of Modeling data

Before first-order seasonal difference				After first-order seasonal difference			
t-Statistic			Prob	t-Statistic			Prob
Augmented Dickey-Fuller test statistic		− 0.13	0.94	Augmented Dickey-Fuller test statistic		−6.09	< 0.01
Test critical values:	1% level	−3.52		Test critical values:	1% level	−3.52	
	5% level	−2.9			5% level	− 2.9	
	10% level	−2.59			10% level	−2.59	

**Fig. 3:**
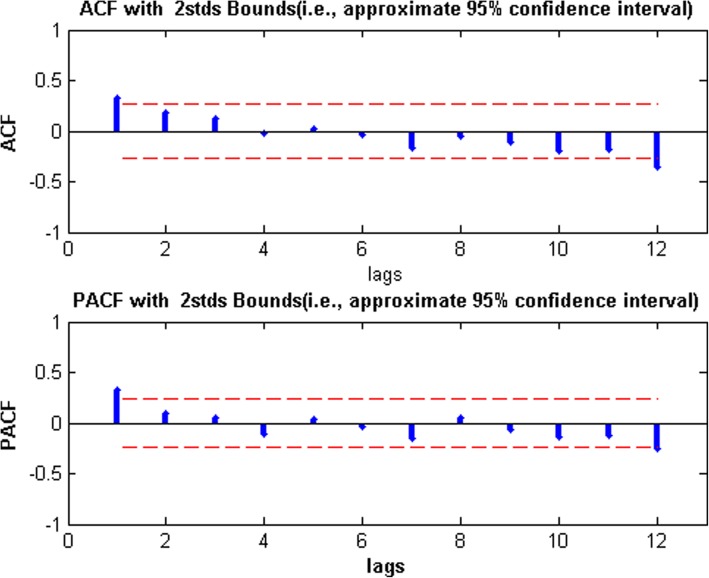
The ACF and PACF graphs of the stationary series

Second, to draw ACF and PACF graphs of stationary data (see Fig. 3). According to the analysis of the ACF and PACF graphs, we established eight tentative models, SARIMA(1,0,1)(0,1,0)_12_,SARIMA(1,0,(2))(0,1,0)_12_,SARIMA((2),0,1)(0,1,0)_12_, SARIMA((2),0,(2))(0,1,0)_12_,SARIMA(2,0,(2))(0,1,0)_12_, SARIMA(2,0,1)(0,1,0)_12_, SARIMA(1,0,2)(0,1,0)_12_, and SARIMA(2,0,2)(0,1,0)_12_. Then, the least square method was used to test the parameters of the eight models, and the AIC and SC values of these models were calculated, the test results were shown in Table 2. It could be seen that only the SARIMA((2),0,(2))(0,1,0)_12_ model with lowest AIC and SC passed the parameter test (all Prob values were less than 0.05).

**Table 2:** The Parameter estimates of the tentative models with their AIC and SC

Model	Variable	Coefficient	Std. Error	t-Statistic	prob	AIC	SC
SARIMA(1,0,1)(0,1,0)_12_	C	−0.81	0.16	−4.92	< 0.01		
	AR(1)	−0.16	0.22	−0.73	0.47	3.00	3.10
	MA(1)	0.51	0.20	2.52	0.01		
SARIMA(1,0,(2))(0,1,0)_12_	C	−0.84	0.21	−4.00	< 0.01		
	AR(1)	0.23	0.12	1.93	0.06	3.03	3.12
	MA(2)	0.27	0.12	2.23	0.03		
SARIMA((2),0,1)(0,1,0)_12_	C	−0.78	0.20	−3.96	< 0.01		
	AR(2)	0.14	0.12	1.21	0.23	2.94	3.04
	MA(1)	0.37	0.12	3.15	< 0.01		
SARIMA((2),0,(2))(0,1,0)_12_	C	−0.77	0.15	−5.22	< 0.01		
	AR(2)	−0.59	0.10	−6.07	< 0.01	2.87	2.98
	MA(2)	0.96	0.02	54.39	< 0.01		
SARIMA(2,0,(2))(0,1,0)_12_	C	−0.78	0.16	−4.83	< 0.01		
	AR(1)	0.17	0.10	1.77	0.08	2.87	3.00
	AR(2)	−0.61	0.10	−6.26	< 0.01		
	MA(2)	0.97	0.02	46.44	< 0.01		
SARIMA(2,0,1)(0,1,0)_12_	C	−0.71	0.22	−3.21	0.00		
	AR(1)	0.64	0.25	2.57	0.01	2.91	3.03
	AR(2)	−0.02	0.14	−0.17	0.86		
	MA(1)	−0.31	0.27	−1.15	0.26		
SARIMA(1,0,2)(0,1,0)_12_	C	−0.81	0.18	−4.46	< 0.01		
	AR(1)	−0.16	0.29	−0.54	0.59	3.01	3.13
	MA(1)	0.50	0.30	1.65	0.10		
	MA(2)	0.18	0.14	1.29	0.20		
SARIMA(2,0,2)(0,1,0)_12_	C	−0.72	0.22	−3.31	< 0.01		
	AR(1)	0.65	0.26	2.52	0.01		
	AR(2)	−0.09	0.21	− 0.44	0.66	2.93	3.09
	MA(1)	−0.33	0.28	−1.17	0.25		
	MA(2)	0.12	0.23	0.52	0.60		

Finally, we did the diagnostic checking of residuals of the SARIMA((2),0,(2))(0,1,0)_12_ model by using the Box-Jenkins Q test, the test Prob was more than 0.05, therefore, according to these analyses, the SARIMA((2),0,(2))(0,1,0)_12_ model was feasible for the prediction of TB incidence in Guangxi.

**Fig. 4:**
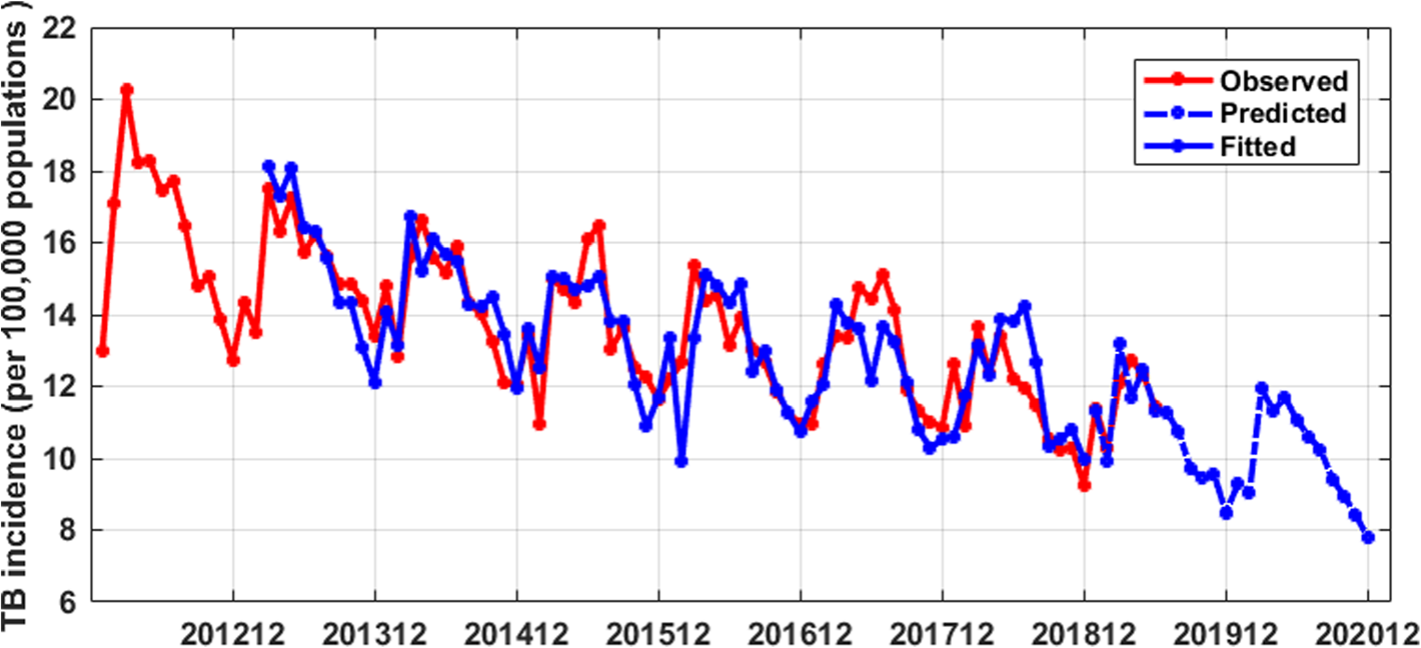
The fitted and predicted TB incidence by the SARIMA((2),0,(2))(0,1,0)_12_ model

We used the SARIMA((2),0,(2))(0,1,0)_12_ model to fit the TB incidence data from March 2013 to December 2018, and the RMSE, MAE, and MAPE were 0.98, 0.77 and 5.8 respectively; We used the SARIMA((2),0,(2))(0,1,0)_12_ model to predict the TB incidence from January 2019 to June 2019, and the RMSE, MAE, and MAPE were 0.62, 0.45 and 3.77, respectively. Both the fitting accuracy values and the prediction accuracy values were very small, which indicated that the SARIMA((2),0,(2))(0,1,0)_12_ model was very good and its prediction accuracy was high. Based on the SARIMA((2),0,(2))(0,1,0)_12_ model, we predicted the TB incidence in Guangxi from July 2019 to December 2020, these predicted values were shown in Table 3, and the fitted and predicted incidence were compared with the observed incidence in Fig. 4.

**Table 3:** The observed TB incidence and predicted TB incidence by SARIMA((2),0,(2))(0,1,0)_12_ model from January 2019 to December 2020

Time	201,901	201,902	201,903	201,904	201,905	201,906
Observed	11.4	10.3	12.12	12.72	12.32	11.45
Predicted	11.34	9.94	13.19	11.71	12.45	11.35
Time	201,907	201,908	201,909	201,910	201,911	201,912
Observed						
Predicted	11.29	10.74	9.73	9.43	9.56	8.48
Time	202,001	202,002	202,003	202,004	202,005	202,006
Observed						
Predicted	9.28	9.05	11.94	11.32	11.68	11.07
Time	202,007	202,008	202,009	202,010	202,011	202,012
Observed						
Predicted	10.61	10.24	9.24	8.95	8.40	7.79

## Discussion

Currently, the annual TB incidence in Guangxi is much higher than that in the national level, although it has been slightly decreasing annually; the potential achievement is diminished by an increasing large-scale transient population, the emergence of MDR-TB, along with the co morbid conditions of AIDS and non-communicable diseases, which have led to a resurgence of TB in recent years [20–22]. Additionally, WHO initiated the End TB Strategy with the target of a 90% reduction in new TB cases by 2035 compared with 2015,and a milestone of reducing the TB incidence by 50% by 2025 relative to 2015 [2], in order to accelerate progress towards such a daunting task, corresponding measures and actions are expected at both the national and international levels. At the national level, every province should make efforts, especially in provinces with high incidence, such as Guangxi. Appropriate plans may fail to be becomingly formulated without getting a clear perspective of the past, current and future temporal levels of this disease, therefore, advanced detection and early response systems for epidemics have formed an integral part of the effective precautions against TB and the reasonable allocation of available health resources.

In this study, the historical trend of TB incidence in Guangxi was carefully analyzed, then, the prediction model of TB incidence in Guangxi was established by using Box Jenkins model method, and this method is one of the most widely used time series forecasting techniques because of its structured modeling basis and acceptable forecasting performance. Through the analysis of the change trend and decomposition graph of original TB incidence, we found that the data had obvious seasonality, trend and randomness, so it is suitable to establish SARIMA model for prediction analysis. For SARIMA model construction, monthly TB incidence from January 2012 to December 2018 was used; for testing the predictive ability of this model, TB incidence from January 2019 to June 2019 was used.

SARIMA model requires data to be stationary, Table 1 showed that the Prob value of ADF test was 0.94 more than 0.05, indicating that the original data was not stable. Considering the seasonal variation of TB incidence data, we did the first-order seasonal difference with a period of 12, after that, we used ADF to test the stationarity of the seasonal-difference data, the Prob value of the test was less than 0.01(see Table 1), which indicated that the difference data was stable and could be used to build SARIMA model. Then, in order to determine the p, q, P and Q in SARIMA(p,0,q)(P,1,Q)_12_ model, the ACF and PACF graphs were drawn, then, eight tentative models were established by the analysis of ACF and PACF graphs. The parameters of these tentative models were tested and these models performance were compared by AIC and SC, Table 2 showed SARIMA((2),0,(2))(0,1,0)_12_ model had smallest AIC and SC, as well as, all the Prob values of its parameter test were less than 0.01, and the Prob value of the Box-Jenkins Q test was more than 0.05, which indicated that the SARIMA((2),0,(2))(0,1,0)_12_ model was feasible to predict the TB incidence in Guangxi. Using SARIMA((2),0,(2))(0,1,0)_12_ model to fit original TB incidence from January 2012 to December 2018, the RMSE(0.98), MAE(0.77), and MAPE(5.80) were very small; Using SARIMA((2),0,(2))(0,1,0)_12_ model to predict TB incidence from January 2019 to June 2019, the RMSE(0.62),MAE(0.45),and MAPE(3.77) were very small too, which indicated that the SARIMA((2),0,(2))(0,1,0)_12_ model was very good and its prediction accuracy was very high. We predicted the TB incidence in Guangxi based on the SARIMA((2),0,(2))(0,1,0)_12_ model from July 2019 to December 2020(see Table 3 and Fig. 4), the results suggested the change trend of predicted TB incidence was similar to change trend in the previous two years, and TB incidence will experience slight decrease, the predicted results can provide scientific reference for the prevention and control of TB in Guangxi, China.

## Conclusions

The incidence of tuberculosis in Guangxi is high, but there is little prediction study of the disease in recent years, advanced detection and early response systems have formed an integral part of the effective precautions against TB and the reasonable allocation of available health resources. In view of this, we used Box-Jenkins method to establish the SARIMA((2),0,(2))(0,1,0)_12_ model for predicting the TB incidence in Guangxi. The RMSE, MAE and MAPE of the SARIMA((2),0,(2))(0,1,0)_12_ were very small, which indicated that the model was successful, its prediction accuracy was high, and its prediction performance was good. Based on SARIMA((2),0,(2))(0,1,0)_12_ model,we predicted the TB incidence of Guangxi from July 2019 to December 2020, the results suggested the TB incidence will experience slight decrease, and its changing trend will be similar to before. The prediction results can provide help for reallocating resources so as to get better in control and prevention of TB in Guangxi, China.

## Supplementary information

**Additional file 1: Table S1.** Monthly TB incidence data from January 2012 to June 2019, Guangxi, China.

## Data Availability

The data used in this study are available from the corresponding author on reasonable request and with permission of the Guangxi center for Disease Control and Prevention, China. The relevant data is provided as Additional file 1.

## Abbreviations

TB: Tuberculosis
ACF: Autocorrelation function;
PACF: Partial autocorrelation function
AIC: Akaike information criterion
SC: Schwarz criterion
SARIMA: Seasonal autoregressive integrated moving average
ADF: Augmented Dickey-Fuller (ADF) test
RMSE: Root mean square error
MAE: Mean absolute error
MAPE: Mean absolute percentage error.

## References

[1] Zhao Y, Li M, Yuan S (2017). Analysis of transmission and control of tuberculosis in mainland China, 2005-2016, based on the age structure mathematical model. Int J Environ Res Public Health.

[2] WHO. Global tuberculosis report 2018. http://www.who.int/tb/publications/global_report/en/. (Accessed on 4 Dec 2018).

[3] Moosazadeh M, Khanjani N, Nasehi M (2015). Predicting the incidence of smear positive tuberculosis cases in Iran using time series analysis. Iran J Public Health.

[4] Tian CW, Wang H, Luo XM (2019). Time-series modelling and forecasting of hand, foot and mouth disease cases in China from 2008 to 2018. Epidemiol Infect.

[5] Wang T, Zhou Y, Wang L (2016). Using an autoregressive integrated moving average model to predict the incidence of hemorrhagic fever with renal syndrome in Zibo, China, 2004-2014. Jpn J Infect Dis.

[6] Gharbi M, Quenel P, Gustave J (2011). Time series analysis of dengue incidence in Guadeloupe, French West Indies: Forecasting models using climate variables as predictors. BMC Infect Dis.

[7] López-Montenegro LE, Pulecio-Montoya AM, Marcillo-Hernández GA (2019). Dengue cases in Colombia: mathematical forecasts for 2018-2022. MEDICC Rev.

[8] Zheng Y-L, Zhang L-P, Zhang X-L (2015). Forecast model analysis for the morbidity of tuberculosis in Xinjiang, China. PLoS ONE.

[9] Liao Z, Zhang X, Zhang Y (2019). Seasonality and Trend Forecasting of Tuberculosis Incidence in Chongqing, China. Interdiscip Sci.

[10] Carvajal Thaddeus M, Viacrusis Katherine M, Hernandez Lara Fides T (2018). Machine learning methods reveal the temporal pattern of dengue incidence using meteorological factors in metropolitan Manila, Philippines. BMC Infect Dis.

[11] Mao Q, Zhang K, Yan W (2018). Forecasting the incidence of tuberculosis in China using the seasonal auto-regressive integrated moving average (SARIMA) model. J Infect Public Health.

[12] Anokye R, Acheampong E, Owusu I, et al. Time series analysis of malaria in Kumasi: Using ARIMA models to forecast future incidence. Cogent Soc Sci. 2018;4(1):1461544.

[13] Withanage GP, Viswakula SD, Yi SGN (2018). A forecasting model for dengue incidence in the district of Gampaha, Sri Lanka. Parasit Vectors.

[14] Siregar FA, Makmur T, Saprin S (2018). Forecasting dengue hemorrhagic fever cases using ARIMA model: a case study in Asahan district. IOP Conference Series Materials Science and Engineering.

[15] Tohidinik HR, Mohebali M, Mansournia MA (2018). Forecasting zoonotic cutaneous leishmaniasis using meteorological factors in eastern Fars province, Iran: a SARIMA analysis. Tropical Med Int Health.

[16] Xu Q, Li R, Liu Y (2017). Forecasting the incidence of mumps in Zibo City based on a SARIMA model. Int J Environ Res Public Health.

[17] Wang H, Tian CW, Wang WM (2018). Time-series analysis of tuberculosis from 2005 to 2017 in China. Epidemiol Infect.

[18] Box GEP, Jenkins GM, Reinsel GC (2015). Time series analysis: forecasting and control, 5th edition. J Oper Res Soc.

[19] Box, George E.P, Jenkins, Gwilym M, Reinsel, Gregory C. Time series analysis. Forecasting and control. 3rd ed. journal of time. 2010;31(4):303.

[20] Moon MS, Kim SS, Moon H (2013). (i) Tuberculosis of the spine: Current views in diagnosis, management, and setting a global standard. Orthopaedics Trauma.

[21] Maitra A, Bates S, Shaik M (2016). Repurposing drugs for treatment of tuberculosis: a role for non-steroidalanti-inflammatory drugs. Br Med Bull.

[22] Berlin L (2008). Tuberculosis: resurgent disease, renewed liability. AJR Am J Roentgenol.

