# Correction to: Statistical methods for predicting tuberculosis incidence based on data from Guangxi, China

**DOI:** 10.1186/s12879-020-05325-8

**Published:** 2020-08-25

**Authors:** Yanling Zheng, Liping Zhang, Lei Wang, Ramziya Rifhat

**Affiliations:** College of Medical Engineering and Technology, Xinjiang Medical University, Urumqi, 830011 People’s Republic of China

**Correction to: BMC Infect Dis 20, 300 (2020).**

10.1186/s12879-020-05033-3

Following publication of the original article [1] the authors made an error in the data source description, described below. To this end, the authors sincerely apologize to Guangxi Center for Disease Control and Prevention, China.

- Incorrect description: The data of the TB cases in Guangxi from January 2012 to June 2019 was obtained from the Guangxi center for Disease Control and Prevention, China (please see Data source section.
- Corrected sentence: The data of the TB cases in Guangxi from January 2012 to June 2019 was obtained from the website of Guangxi Health Management Committee (http://wsjkw.gxzf.gov.cn/zfxxgk_49572/ggws/fdcrbyqgb/t5518731.shtml).

The following sections have also been revised accordingly:

**Availability of data and materials.**

The data used in this study are available from the corresponding author on reasonable request. The relevant data is provided as Additional file 1.

**Ethics approval and consent to participate.**

The research did not involve any direct participation by human subjects. The TB data were extracted from monthly reports maintained on public website (http://wsjkw.gxzf.gov.cn/zfxxgk_49572/ggws/fdcrbyqgb/t5518731.shtml).
